# Artificial Intelligence in Perioperative Planning and Management of Liver Resection

**DOI:** 10.1007/s13193-024-01883-4

**Published:** 2024-01-23

**Authors:** Shruti Gairola, Sohan Lal Solanki, Shraddha Patkar, Mahesh Goel

**Affiliations:** 1 Department of Anaesthesiology, Critical Care and Pain, Tata Memorial Hospital, Homi Bhabha National Institute, Mumbai, Maharashtra India; 2 Division of Hepatobiliary Surgical Oncology, Department of Surgical Oncology, Tata Memorial Hospital, Homi Bhabha National Institute, Mumbai, Maharashtra India

**Keywords:** Artificial intelligence, Perioperative management, Liver resections

## Abstract

Artificial intelligence (AI) is a speciality within computer science that deals with creating systems that can replicate the intelligence of a human mind and has problem-solving abilities. AI includes a diverse array of techniques and approaches such as machine learning, neural networks, natural language processing, robotics, and expert systems. An electronic literature search was conducted using the databases of “PubMed” and “Google Scholar”. The period for the search was from 2000 to June 2023. The search terms included “artificial intelligence”, “machine learning”, “liver cancers”, “liver tumors”, “hepatectomy”, “perioperative” and their synonyms in various combinations. The search also included all MeSH terms. The extracted articles were further reviewed in a step-wise manner for identification of relevant studies. A total of 148 articles were identified after the initial literature search. Initial review included screening of article titles for relevance and identifying duplicates. Finally, 65 articles were reviewed for this review article. The future of AI in liver cancer planning and management holds immense promise. AI-driven advancements will increasingly enable precise tumour detection, location, and characterisation through enhanced image analysis. ML algorithms will predict patient-specific treatment responses and complications, allowing for tailored therapies. Surgical robots and AI-guided procedures will enhance the precision of liver resections, reducing risks and improving outcomes. AI will also streamline patient monitoring, better hemodynamic management, enabling early detection of recurrence or complications. Moreover, AI will facilitate data-driven research, accelerating the development of novel treatments and therapies. Ultimately, AI’s integration will revolutionise liver cancer care, offering personalised, efficient and effective solutions, improving patients’ quality of life and survival rates.

## Introduction

### Basic Concepts and History of Artificial Intelligence

Though artificial intelligence (AI) is considered a recent development, it has origin to way back in history. The term artificial intelligence was coined by John McCarthy in 1956. He defined it as “the science and engineering of making intelligent machines” [1]. The history of AI in healthcare dates back to 1960–1970s where researches began to explore the potential of AI in medical diagnosis in healthcare. Programmes like MYCIN focussed on diagnosing bacterial infections. MYCIN had the capability to identify bacteria causing severe infections and recommend specific antibiotic treatments [2]. In the 1980–1990s, systems like CADUCEUS and INTERNIST demonstrated the ability to provide diagnostic information based on the patients’ medical data and the medical knowledge. By 2000s, data mining and pattern recognition started being applied to large datasets in order to identify disease trends and predicting outbreaks. In the year 2010, machine learning and particularly deep learning revolutionised medical imaging analysis such as detecting diseases from medical images from the X-rays, MRI and CT scans.

### What Is AI?

It is a speciality within computer science that deals with creating systems that can replicate the intelligence of a human mind and has problem-solving abilities. AI includes a diverse array of techniques and approaches such as machine learning, neural networks, natural language processing, robotics, and expert systems. There are 3 stages of AI:

- Narrow or weak AI which is trained for a specific task like chatbots,
- General or strong AI which possesses human-like cognitive abilities and can theoretically perform any intellectual task that a human being can do.
- Super AI alludes to a stage when computers would surpass human capabilities.

Machine learning (ML) is a subset of AI techniques which involves programmes that allow computers to learn from examples and patterns from data and make predictions of decisions based without being explicitly programmed for a specific scenario. Machine learning can be categorised into supervised learning (models are trained on labelled data), unsupervised learning (machine trains with unlabelled data) and reinforcement learning (models learn through trial and error based on rewards and penalties).

In recent times, ML, which falls under the umbrella of AI, has triggered a significant transformation across various domains in the medical field, paving the way for innovative advancements in hepatic surgery. The remarkable progress of ML in medicine has been facilitated by the existence of extensive datasets and the enhancement of computing capabilities. ML operates as a computer-driven technique that automates the construction of analytical models. [3].

ML is characterised by the discovery and testing of algorithms that help on the recognition, classification and prediction of patterns, all of which are derived from models constructed using pre-existing data [4].

Deep learning (DL) is a subfield of machine learning. It is inspired by how neural network of a human brain is designed structurally and functionally. DL involves training artificial neural networks (ANNs) to perform tasks by learning from large amounts of data sets. This neural network comprises of multiple hidden layers between the input and output layers. The key components of DL comprises of neural networks, activation function, backpropagation, gradient descent and deep architecture [5].

Natural language processing (NLP) is a technique used in text mining and represents a facet of AI. It revolves around human languages, enabling computers to comprehend and interpret human-written texts. A few applications of NLP include auto-correction, autocompletion in searches, etc.

The Generative Pre-trained Transformer (GPT-3) stands as an autoregressive language model that utilises deep learning to produce human-like text [6].

### AI in Healthcare

The use of AI in healthcare presently revolves around diagnosis, treatment recommendation, patient engagement and adherence and administrative activities [7–10]. With the availability of large amounts of clinical data in electronic health records (EHRs), Picture Archiving and Communication Systems (PACS) have enabled the use of AI in various fields of medicine [11].

The influence of AI will extend across all realms of medicine, including preclinical and clinical areas, resulting in a comprehensive transformation of the entire healthcare landscape.

IBM’s tool “Watson for Oncology” is one example of a system that provides oncologists treatment recommendations [12, 13]. The treatment suggestions provided by both the Manipal Multidisciplinary Tumour Board and Watson for Oncology exhibited strong agreement in the studied cancer cases [14, 15].

Streams, an AI-powered app for early detection of acute kidney injury, was developed in 2016. In 2021, DeepMind’s AlphaFold in Biology made significant strides in predicting protein structures, which has implications for understanding diseases and drug design in the field of biology. [16].

In April 2018, the US Food and Drug Administration granted approval for the first software system driven by AI. This programme aids in diagnosing diabetic retinopathy by analysing fundus images.

As AI continues to evolve, it has increasingly found its use in healthcare. Conversely, numerous advancements in AI owe their origins and inspiration to disciplines such as biology, psychology, and the broader domain of medical sciences. Artificial neuron, insights from the visual cortex led to development of neocognitron paving way for convolutional neural network and triggering the era of DL in AI.

AI has the potential to have an impact in various aspects of anaesthesia care extending from preoperative planning, assessment and evaluation to intraoperative administration of care and extending to the postoperative period as part of critical care delivery and pain management.

## Methods

### Literature Search Strategy

An electronic literature search was conducted using the databases of “PubMed” and “Google Scholar”’ The period for the search was from 2000 to June 2023. The search terms included “artificial intelligence”, “machine learning”, “liver cancers”, “liver tumors”, “hepatectomy”, “perioperative” and their synonyms in various combinations. The search also included all MeSH terms. The extracted articles were further reviewed in a step-wise manner for identification of relevant studies. The titles and abstracts were inspected independently by two authors (SG and SLS).

### Study Selection Criteria

All the articles including review article, observational studies or control trials and published in English language, having full texts and described use of AI in liver cancer surgeries were reviewed. Articles without an abstract or full-text were excluded.

### Literature Search Results

A total of 148 articles were identified after the initial literature search. Initial review included screening of article titles for relevance and identifying duplicates. Finally, 65 articles were reviewed for this review article.

## Artificial Intelligence in Liver Resection

### Preoperative Planning and Optimisation

AI is increasingly utilised in areas such as risk assessment, genomics, imaging and diagnostics, precision medicine and the exploration of new pharmaceuticals. The use of AI in liver resection surgeries and hepatology is relatively new (Table 1). Liver resection is an important treatment modality for cancers of the liver but it is challenging due to complex anatomy of liver and concerns about functional liver remnant (FLR). Surgical technique involves excising the diseased part with adequate oncological clearance ensuring minimal blood loss and leaving behind enough healthy liver to avoid postoperative liver failure. AI has been used to enhance intraoperative visualisation of preoperative imaging data in hepatic surgeries by focussing on imaging and navigation. 3D visualisation techniques and 3D printing technology have aided in the visualisation of intricate anatomical details of the liver during complex resections [17]. Using the 3D printing technique, virtual liver resection can be performed to assess the respectability and calculate the FLR [18]. ML has found applications across multiple facets of the 3D printing process, enhancing the entirety of the design and manufacturing workflow.

**Table 1:** Artificial intelligence in liver resection

1	Preoperative planning and optimization
	Assessment of FLR
	1.2 Assessment of risk of Post Hepatectomy Liver Failure (PHLF)
	1.3 Prediction of oncological outcome
	1.4 Predicting tumour response to TACE therapy
2	AI in anaesthesia for liver resection
	2.1 Preoperative – • Perioperative Risk Stratification – Predictive OpTimisation Trees in Emergency Surgery Risk (POTTER), MySurgeryRisk Score • Detection of difficult airway
	2.2 Intraoperative • Prediction of intraoperative hypotension • Monitoring depth of anaesthesia • Closed-loop delivery system • Automation of fluid therapy • Automation of vasopressor titration and inotrope infusion • AI in regional anaesthesia – ultrasound guidance
	2.3 Postoperative • Pain assessment and management • Venous thromboembolism • Predicting length of hospital stay, prediction of postoperative complications

Various AI-based algorithms have been used for assessment of FLR also known as automated hepatic volumetry. Image segmentation algorithms (analyse medical images like CT scans and MRI to calculate FLR), volumetric analysis algorithms, blood vessel detection algorithms, functional assessment algorithms, risk prediction algorithms, etc. collectively contribute to the accurate assessment of the future liver remnant. Wang et al. have introduced a convolutional neural network (CNN) algorithm designed to entirely automate the assessment of liver volume using both CT and MRI data. [19] Similarly, Winkel et al. have devised an algorithm that exhibits good precision, rapid processing and strong concordance with manual segmentation [20].

Several scoring systems have been used to predict the quality of the liver and risk of post hepatectomy liver failure (PHLF) like the Child–Pugh grade, modified end-stage liver disease (MELD) score, albumin-bilirubin (ALBI), platelet-albumin-bilirubin (PALBI), the aspartate aminotransferase to platelet ratio index (APRI) and the fibrosis index (FIB-4) [21–25].

Mai et al. designed an ANNs model, encompassing blood tests (platelet count, total bilirubin, prothrombin time and aspartate aminotransferase) and anticipated FLR. It demonstrated better accuracy in predicting severe PHLF, with an area under the curve (AUC) value of 0.880, surpassing existing predictive systems [26].

The recurrence rate after liver resection surgeries remains high. At present, there are no reliable risk stratification models for cancer recurrence after liver resection. Researchers have examined the prediction of disease-free survival (DFS) at 1, 3 and 5 years for patients with hepatocellular carcinoma undergoing liver resection through the application of artificial neural networks. One such model studied by Ho et al. exhibited superior performance compared to the conventional logistic regression models for prediction accuracy [27].

Saillard et al. developed a prognostic model for predicting survival in patients with hepatocellular carcinoma (HCC) who underwent liver resection. They constructed a DL model using HCC digital slides, which demonstrated greater accuracy in comparison to primary scoring systems encompassing clinical, biological and pathological characteristics [28].

Transarterial chemoembolization (TACE) is an important treatment for intermediate stage HCC. However, the response of HCC to this therapy is extremely variable. Currently, serological biomarkers and magnetic resonance imaging (MRI) are used to predict response to TACE. Scorings systems such as HAP score (the hepatoma arterial embolization prognostic) and STATE score (the selection for TACE treatment) have been used to predict outcomes in patients of HCC undergoing TACE. Dynamic contrast-enhanced ultrasound (CEUS) cines can better trace microcirculation perfusion of tumours and thus have been employed in AI-based radiomics strategies. Liu et al. enrolled 130 patients undergoing TACE treatment. Special radiomics models, employing both deep learning (DL) and machine learning (ML) techniques, were developed and trained using dynamic contrast-enhanced ultrasound (CEUS) cine images for dynamic features and static B-mode images for static features. These models were designed to predict personalised tumour responses to TACE therapy. They found that AI analysis tool was much more efficient than the existing HAP score in predicting prognosis of TACE [29].

In the realm of perioperative medicine, AI models have been used for stratifying perioperative risks, monitoring patients during surgery and managing intensive care [30].

### AI in Anaesthesia for Liver Resection

#### Perioperative Risk Stratification

Perioperative risk stratification is important to facilitate shared decision-making amongst the clinician, patient and care givers. Numerous assessments have been employed previously including the American Society of Anesthesiologists Physical Status (ASA-PS) classification, American College of Surgeons National Surgical Quality Improvement Program (ACS-NSQIP) surgical risk calculator and Risk Stratification Index [31]. Recently, AI models for perioperative risk stratification have been shown to predict accurately risk of postoperative complications.

Various AI and ML platforms have been developed which gather vast amount of data from the EHRs and can more accurately predict risks. The Predictive OpTimization Trees in Emergency Surgery Risk (POTTER) calculator is a ML platform that utilises data from the ACS-NSQIP database. It is a surgical risk calculator which predicts postoperative 30 days mortality and 18 postoperative complications [32]. Its performance was found to be stable when tested in patients undergoing emergency general surgery and laparotomy [33].

The most commonly used model for prediction of cardiac risk is Lee’s Revised Cardiac Risk Index (RCRI) [34]. However, a systematic review of 24 studies showed only a moderate level of performance [35]. MySurgeryRisk score is another preoperative risk algorithm that employs clinical data from EHRs and calculates probabilistic risk scores for 8 significant postoperative complications (acute kidney injury, sepsis, venous thromboembolism, intensive care unit admission > 48 h, mechanical ventilation > 48 h, wound, neurologic, and cardiovascular complications) and mortality up to 24 months after surgery. The AUC values for these predictions range from 0.82 to 0.94 [36].

PHLF is a dreaded complication post liver resection surgeries. Various scoring systems have been used in the past, namely FIB-4, APRI, CTP score, MELD and ALBI for evaluation of liver function and predicting PHLF. Various inflammatory markers like albumin globulin ratio (AGR), neutrophil to lymphocyte ratio (NLR), platelet to lymphocyte ratio (PLR) and aspartate transaminase to platelet ratio index (APRI) have been used to predict immediate postoperative complications [37]. However, the predictive accuracy of these tools is poor in comparison to AI-based combined models. Mai and colleagues devised an artificial neural network–based model to predict the risk of PHLF in patients with HCC who were undergoing partial hepatectomy [26]. The ML PHLF model is one such AI-based model that utilises 25 variables and has good predictive value for AHLF [38].

The use of AI in predicting events related to peri-anaesthetic care can be broadly divided into:

Preoperative domain—predicting the class of anaesthetic risk, detection of difficult intubation, etc.

Intraoperative optimisation—prediction of depth of anaesthesia in response to propofol boluses, prediction of duration of recovery from the effect of neuromuscular blockade, etc.

Postoperative period—prediction of duration of recovery or length of hospital stay, prediction of postoperative complications, deterioration, ICU stay or mortality.

Airway-related concerns—computerised facial analysis has been used for the accurate classification of difficult intubation. The best computer model included 3 facial parameters and thyromental distance and correctly classified 70 out of 80 patients studied. This was significantly better than the popular clinical predictive tests [39]. In a study, 1000 patients undergoing elective surgery were evaluated using semi-supervised DL method for identifying difficulties of both mask ventilation and intubation. This model demonstrated better performance as compared to the conventional methods of prediction of difficult airway [40].

#### Prediction of Intraoperative Hypotension

Kendale et al. formulated a machine learning model for prediction of postinduction hypotension (defined as mean arterial pressure below 55 mmHg within 10 min of induction time) using information available from the EHR. Gradient Boosting Machine (GBM) algorithm demonstrated superior performance compared to other algorithms [41]. Hatib et al. developed a machine learning algorithm using thousands of arterial waveform known as hypotension prediction index (HPI) to predict intraoperative hypotensive events (MAP < 65 mmHg) 15 min before its occurrence with a sensitivity of 88% and specificity of 87% [42]. Gangakhedkar et al. in a case series reported the use of HPI in patients undergoing cytoreductive surgery (CRS) with hyperthermic intraperitoneal chemotherapy (HIPEC). They found it useful to predict hypotension and alert surgeons to a concealed source of bleeding on 2 occasions during the CRS phase [43].

Lee et al. in their retrospective observational analysis obtained data from 3301 patients. The researchers utilised deep learning algorithms to create real-time predictions for impending intraoperative hypotensive events at 5, 10 and 15 min using biosignal waveforms in noncardiac surgical scenarios. These models were created to carry out tasks involving classification and regression. Classification model was a binary classifier of a hypotensive (MAP, 65 mmHg lasting more than 1 min) or non-hypotensive event. The regression model estimated the MAP as a continuous value for 5, 10 and 15 min. The main result measured was the area under the receiver-operating characteristic (AUROC) curve for the classification models and the mean absolute error (MAE) for the regression models. This model demonstrated optimal performance across wide range of patients and surgical procedures [44].

#### Monitoring Depth of Anaesthesia

Majority of literature in AI for anaesthesia comes from techniques trying to find depth of anaesthesia (DOA). Utilising neural network analysis for categorising electroencephalographic (EEG) patterns in relation to the level of midazolam sedation amongst patients in the intensive care unit dates back to 1991 [45].

These DL models produce a value of awareness using the regression model [46] or use a binary classification to classify patient as awake or unconscious [47] or use multi-label classification models [48]. Data such as EEG signals can be well analysed by ML techniques. Several studies have compared the performance of ML models with the conventional anaesthesia depth monitors such as bispectral index [49]. More recent studies have used raw EEGs data for AI input to more directly estimate the depth of anaesthesia. Other range of signals have also been used such as heart rate variability [50], auditory evoked potentials, vital signs and end-tidal carbon dioxide.

Solanki et al., in their narrative review on the application of AI in the perioperative management of major gastrointestinal surgeries, highlighted that achieving the appropriate depth of anaesthesia (DOA) is a delicate equilibrium that depends on the careful balance between administering anaesthesia agents and continuously monitoring relevant parameters. Any deviation, whether it leads to under-dosing or excessive DOA, can happen due to equipment imbalances. Consequently, the field of automated anaesthesia is still considered to be in its early stages of development [51].

#### Controlling Anaesthesia Delivery with AI

Closed-loop delivery system can be used to administer various anaesthetic drugs, intravenous fluids and vasopressors. It requires an input that can be a measurement of depth of anaesthesia from EEG signal, a nociception monitor or some hemodynamic parameters, etc. This can be achieved using simple pharmacokinetic models or using machine learning algorithms. Moore et al. in their study on reinforced learning-based propofol-induced hypnosis in healthy human volunteers showed greater accuracy and stability when compared to conventional technique [52]. Yang et al. compared amongst 70 patients undergoing major abdominal surgeries between closed-loop target control infusion(CLTCI) and open-loop target control infusion (OLTCI). They found that the percentage of adequate anaesthesia time at induction was higher in the CLTCI group. During the maintenance stage, the percentage of deep anaesthesia time was significantly lower in the CLTCI infusion group [53]. A multicentre randomised controlled trial to evaluate closed-loop anaesthesia delivery system looked at 242 patients. The study revealed that the CLADS group maintained BIS within ± 10 of the target for a considerably longer duration compared to the manual group [54].

#### Automation for Fluid Therapy, Vasopressor Titration and Ionotrope Infusion

A scoping review looking at closed-loop controlled fluid administration systems identified ten such distinct management systems, across different stages of development and for numerous intended purposes [55]. ML and complex feature extraction techniques make use of the unique dynamic signatures in the arterial waveform that enable prediction of hypotensive events [56, 57]. In one of the earlier studies conducted in 2013 is major aortic vascular and hepatobiliary surgery to provide acceptable goal-directed fluid therapy. They used a closed-loop algorithm that was thoroughly evaluated before it was used in the clinical setting. It was also tested in the criterial for compliance with intraoperative goal-directed fluid therapy [58].

In a case–control study amongst patients undergoing high-risk hepatobiliary surgeries comparing closed-loop assisted versus manual goa -directed fluid therapy (GDFT), closed-loop group had a significantly greater duration of case time in a preload-independent state and also showed significantly lower case mean SVV. This study demonstrates that the closed-loop fluid administration system has the potential to aid healthcare professionals in delivering and potentially enhancing the quality of GDFT [59].

Similarly, closed-loop vasopressor controllers have also been studied for their feasibility in clinical settings. A RCT of 30 patients undergoing major abdominal surgeries is randomised into 2 groups: closed-loop vasopressor (CLV) vs manually controlled group. They found that in the CLV group, the occurrence of hypotension during surgery was one-tenth of that observed in the control group [60].

Another RCT studied 38 patients undergoing abdominal and orthopedic surgeries divided into a computer-assisted individualised hemodynamic group and manually adjusted GDFT group where the regulation of vasopressor and fluid administration was carried out manually by the anaesthesia providers to study intraoperative hypotension. The computer-assisted group showed a lower hypotension rate (MAP less than 90% of their baseline) at 1.2%. This was in contrast to the manually adjusted GDFT group, which exhibited a considerably higher rate of 21.5% [61].

#### AI in Regional Anaesthesia

Ultrasound guidance—ultrasound has been used for identification of vertebral level and other anatomical landmarks for placement of epidural catheters, identification of vessels, etc. Researchers have made use of the convolutional neural networks to automate identification of various vertebral levels [62, 63]. Zhao et al. have developed an artificial neural network (ANN) to automatically detect (in real time) anatomical structures within ultrasound images of thoracic paravertebral block (TPVB). The accuracy rates of the Paravertebral space, lung and bone were 91.7%, 95.4% and 74.3%, respectively [64]. Devices driven by AI have the potential to assist in both the acquisition and interpretation of images in ultrasound-guided regional anaesthesia (UGRA). The ScanNav™ (Intelligent Ultrasound, Cardiff, UK) is an artificial intelligence–based device that produces a colour overlay on real-time ultrasound images to highlight anatomical structures of interest. A study by Bowness et al found that ScanNav™ could identify anatomical structures important for a safe and efficacious ultrasound-guided regional anaesthesia in 93.5% cases. It was estimated that this could bring down the risk of recognised complications in 62.9–86.2% of scans and reduce the rate of block failure in 81.2% of scans as per subjective expert opinion [65]. Several convolutional neural networks (CNN) have been developed to facilitate nerve tracking in UGRA [66]. The automated spinal landmark identification technique was studied in 100 patients requiring spinal anaesthesia in terms of the success rate of spinal anaesthesia at the first attempt. The success rate was found to be 92% with the median time for detection of the posterior complex around 45 s [67].

Pain management—AI has been variously used in assessment and management of pain. Major development in the use of AI in the domain of pain management had happened over the last 10 years. Various studies have focussed on the AI-based assessment of pain. Many of these models for automated pain assessment were based on facial expressions [68]. A study comparing artificial neural network against an established scoring system for classification of patients based on neuropathic pain symptoms showed only a slightly higher accuracy with the former [69].

AI-based models can also be used for pain prediction and this can be useful in the postoperative period. AI can also be used for opioid dosing. A sequential decision-making framework for opioid dosing based on deep reinforcement learning demonstrates that reinforcement learning can be used to help in opioid dosing [70].

#### AI and VTE

AI has been utilised in diagnosing and predicting VTE in recent studies. In a recent meta-analysis done by Wang et al. looking at 12 studies found AI to help in diagnosing and predicting venous thrombosis [71]. Naseem et al. found the incidence of VTE 32.55 per 100,000 admission per year [72]. So we see that the incidence of VTE is not low but the diagnostic accuracy of the current tests available is not high. Various AI research methods like natural language processing (NLP), artificial neural networks (ANNs) and support vector machines (SVMs) have been used for prediction and diagnosis of VTE. Zhou et al. found that the incidence of VTE was reduced by 19.35% after implementing Artificial Intelligence–based Clinical Decision Support System** (**AI-CDSS). They also found the use of anticoagulant drugs which was 22.88% in the intervention group, increased by 14.57% after implementing AI-CDSS [73].

## Bioethics in Artificial Intelligence

Bioethics plays a crucial role in the integration of AI with medical sciences. Firstly, patient consent and privacy are central to bioethics. It is important that patients must be informed about the use of AI in their treatment planning and optimization, its potential benefits, and any accompanying risks. They should have the autonomy to choose whether to proceed with AI-assisted planning and management or opt for conventional methods [74, 75].

Another critical aspect is transparency and accountability. Healthcare professionals and researchers must ensure that AI algorithms used in liver resection are transparent, well-documented and continually monitored for biases or errors. Patients and surgeons should have confidence in the technology’s accuracy and fairness [74, 75].

Additionally, ethical frameworks should address issues of data ownership, as AI relies on vast datasets for training. Protecting patient data, obtaining proper consent for data usage and preventing misuse are ethical imperatives [74, 75].

Lastly, ongoing evaluation and regulation are critical to ensuring that AI remains ethical. Regulatory bodies should oversee the development and deployment of AI systems, ensuring compliance with ethical standards and safety measures.

## Future of Artificial Intelligence

The future of AI in liver cancer planning and management holds immense promise. AI-driven advancements will increasingly enable precise tumour detection, location and characterisation through enhanced image analysis. ML algorithms will predict patient-specific treatment responses and complications, allowing for tailored therapies. Surgical robots and AI-guided procedures will enhance the precision of liver resections, reducing risks and improving outcomes. AI will also streamline patient monitoring, better hemodynamic management, enabling early detection of recurrence or complications. Moreover, AI will facilitate data-driven research, accelerating the development of novel treatments and therapies. Ultimately, AI’s integration will revolutionise liver cancer care, offering personalised, efficient and effective solutions, improving patients’ quality of life and survival rates.

## References

[1] Hamet P, Tremblay J (2017) Artificial intelligence in medicine. Metabolism 69S:S36–S40. 10.1016/j.metabol.2017.01.011 28126242 10.1016/j.metabol.2017.01.011

[2] Perry CA (1990) Knowledge bases in medicine: a review. Bull Med Libr Assoc 78(3):271–282 2203499 PMC225405

[3] Baxt WG (1995) Application of artificial neural networks to clinical medicine. Lancet 346(8983):1135–1138. 10.1016/s0140-6736(95)91804-3 7475607 10.1016/s0140-6736(95)91804-3

[4] Tarca AL, Carey VJ, Chen XW, Romero R, Drăghici S (2007) Machine learning and its applications to biology. PLoS Comput Biol 3(6):e116. 10.1371/journal.pcbi.0030116 17604446 10.1371/journal.pcbi.0030116 PMC1904382

[5] Vakalopoulou M, Christodoulidis S, Burgos N, Colliot O, Lepetit V (2023) Deep Learning: Basics and Convolutional Neural Networks (CNNs). In: Colliot O, editor. Machine Learning for Brain Disorders. New York, NY: Humana; 77–115. 10.1007/978-1-0716-3195-9_3 37988533

[6] Khan RA, Jawaid M, Khan AR, Sajjad M (2023) ChatGPT - Reshaping medical education and clinical management. Pak J Med Sci 39(2):605–607. 10.12669/pjms.39.2.7653 36950398 10.12669/pjms.39.2.7653 PMC10025693

[7] Davenport T, Kalakota R (2019) The potential for artificial intelligence in healthcare. Future Healthc J 6(2):94–98. 10.7861/futurehosp.6-2-94 31363513 10.7861/futurehosp.6-2-94 PMC6616181

[8] Thrall JH, Li X, Li Q et al (2018) Artificial intelligence and machine learning in radiology: opportunities, challenges, pitfalls, and criteria for success. J Am Coll Radiol 15(3 Pt B):504–508. 10.1016/j.jacr.2017.12.026 29402533 10.1016/j.jacr.2017.12.026

[9] Salto-Tellez M, Maxwell P, Hamilton P (2019) Artificial intelligence-the third revolution in pathology. Histopathology 74(3):372–376. 10.1111/his.13760 30270453 10.1111/his.13760

[10] Hashimoto DA, Rosman G, Rus D, Meireles OR (2018) Artificial intelligence in surgery: promises and perils. Ann Surg 268(1):70–76. 10.1097/SLA.0000000000002693 29389679 10.1097/SLA.0000000000002693 PMC5995666

[11] Topol EJ (2019) High-performance medicine: the convergence of human and artificial intelligence. Nat Med 25(1):44–56. 10.1038/s41591-018-0300-7 30617339 10.1038/s41591-018-0300-7

[12] Zhou N, Zhang CT, Lv HY et al (2019) Concordance study between IBM Watson for oncology and clinical practice for patients with cancer in China. Oncologist 24(6):812–819. 10.1634/theoncologist.2018-0255 30181315 10.1634/theoncologist.2018-0255 PMC6656482

[13] Doyle-Lindrud S (2015) Watson will see you now: a supercomputer to help clinicians make informed treatment decisions. Clin J Oncol Nurs 19(1):31–32. 10.1188/15.CJON.31-32 25689646 10.1188/15.CJON.31-32

[14] Liu C, Liu X, Wu F, Xie M, Feng Y, Hu C (2018) Using artificial intelligence (Watson for Oncology) for treatment recommendations amongst Chinese patients with lung cancer: feasibility study. J Med Internet Res 20(9):e11087. 10.2196/11087. (Published 2018 Sep 25) 30257820 10.2196/11087 PMC6231834

[15] Tian Y, Liu X, Wang Z et al (2020) Concordance between Watson for Oncology and a multidisciplinary clinical decision-making team for gastric cancer and the prognostic implications: retrospective study. J Med Internet Res. 22(2):e14122. 10.2196/14122. (Published 2020 Feb 20) 32130123 10.2196/14122 PMC7059081

[16] Jumper J, Evans R, Pritzel A et al (2021) Highly accurate protein structure prediction with AlphaFold. Nature 596(7873):583–589. 10.1038/s41586-021-03819-2 34265844 10.1038/s41586-021-03819-2 PMC8371605

[17] Yang T, Lin S, Xie Q et al (2019) Impact of 3D printing technology on the comprehension of surgical liver anatomy. Surg Endosc 33(2):411–417. 10.1007/s00464-018-6308-8 29943060 10.1007/s00464-018-6308-8

[18] Mise Y, Hasegawa K, Satou S et al (2018) How has virtual hepatectomy changed the practice of liver surgery?: Experience of 1194 virtual hepatectomy before liver resection and living donor liver transplantation. Ann Surg 268(1):127–133. 10.1097/SLA.0000000000002213 28288065 10.1097/SLA.0000000000002213

[19] Wang K, Mamidipalli A, Retson T, Bahrami N, Hasenstab K, Blansit K, Bass E, Delgado T, Cunha G, Middleton MS, Loomba R, Neuschwander-Tetri BA, Sirlin CB, Hsiao A, Members of the NASH Clinical Research Network (2019) Automated CT and MRI liver segmentation and biometry using a generalized convolutional neural network. Radiol Artif Intell 1:180022. 10.1148/ryai.2019180022 32582883 10.1148/ryai.2019180022 PMC7314107

[20] Winkel DJ, Weikert TJ, Breit HC et al (2020) Validation of a fully automated liver segmentation algorithm using multi-scale deep reinforcement learning and comparison versus manual segmentation. Eur J Radiol 126:108918. 10.1016/j.ejrad.2020.108918 32171914 10.1016/j.ejrad.2020.108918

[21] Veerankutty FH, Jayan G, Yadav MK et al (2021) Artificial Intelligence in hepatology, liver surgery and transplantation: emerging applications and frontiers of research. World J Hepatol 13(12):1977–1990. 10.4254/wjh.v13.i12.1977 35070002 10.4254/wjh.v13.i12.1977 PMC8727218

[22] Durand F, Valla D (2005) Assessment of the prognosis of cirrhosis: Child-Pugh versus MELD. J Hepatol 42(1):S100–S107. 10.1016/j.jhep.2004.11.015 15777564 10.1016/j.jhep.2004.11.015

[23] Johnson PJ, Berhane S, Kagebayashi C et al (2015) Assessment of liver function in patients with hepatocellular carcinoma: a new evidence-based approach-the ALBI grade. J Clin Oncol 33(6):550–558. 10.1200/JCO.2014.57.9151 25512453 10.1200/JCO.2014.57.9151 PMC4322258

[24] Lu LH, Zhang YF, Mu-Yan C et al (2019) Platelet-albumin-bilirubin grade: risk stratification of liver failure, prognosis after resection for hepatocellular carcinoma. Dig Liver Dis 51(10):1430–1437. 10.1016/j.dld.2019.04.006 31054962 10.1016/j.dld.2019.04.006

[25] Zhou P, Chen B, Miao XY et al (2020) Comparison of FIB-4 index and Child-Pugh Score in predicting the outcome of hepatic resection for hepatocellular carcinoma. J Gastrointest Surg 24(4):823–831. 10.1007/s11605-019-04123-1 31066014 10.1007/s11605-019-04123-1

[26] Mai RY, Lu HZ, Bai T et al (2020) Artificial neural network model for preoperative prediction of severe liver failure after hemihepatectomy in patients with hepatocellular carcinoma. Surgery 168(4):643–652. 10.1016/j.surg.2020.06.031 32792098 10.1016/j.surg.2020.06.031

[27] Ho WH, Lee KT, Chen HY, Ho TW, Chiu HC (2012) Disease-free survival after hepatic resection in hepatocellular carcinoma patients: a prediction approach using artificial neural network. PLoS ONE 7(1):e29179. 10.1371/journal.pone.0029179 22235270 10.1371/journal.pone.0029179 PMC3250424

[28] Saillard C, Schmauch B, Laifa O et al (2020) Predicting survival after hepatocellular carcinoma resection using deep learning on histological slides. Hepatology 72(6):2000–2013. 10.1002/hep.31207 32108950 10.1002/hep.31207

[29] Liu D, Liu F, Xie X et al (2020) Accurate prediction of responses to transarterial chemoembolization for patients with hepatocellular carcinoma by using artificial intelligence in contrast-enhanced ultrasound. Eur Radiol 30(4):2365–2376. 10.1007/s00330-019-06553-6 31900703 10.1007/s00330-019-06553-6

[30] Hashimoto DA, Witkowski E, Gao L, Meireles O, Rosman G (2020) Artificial intelligence in anesthesiology: current techniques, clinical applications, and limitations. Anesthesiology 132(2):379–394. 10.1097/ALN.0000000000002960 31939856 10.1097/ALN.0000000000002960 PMC7643051

[31] Bilimoria KY, Liu Y, Paruch JL et al (2013) Development and evaluation of the universal ACS NSQIP surgical risk calculator: a decision aid and informed consent tool for patients and surgeons. J Am Coll Surg 217(5):833–42.e423. 10.1016/j.jamcollsurg.2013.07.385 24055383 10.1016/j.jamcollsurg.2013.07.385 PMC3805776

[32] Bertsimas D, Dunn J, Velmahos GC, Kaafarani HMA (2018) Surgical risk is not linear: derivation and validation of a novel, user-friendly, and machine-learning-based Predictive OpTimal Trees in Emergency Surgery Risk (POTTER) Calculator. Ann Surg 268(4):574–583. 10.1097/SLA.0000000000002956 30124479 10.1097/SLA.0000000000002956

[33] El Hechi MW, Maurer LR, Levine J et al (2021) Validation of the artificial intelligence-based Predictive Optimal Trees in Emergency Surgery Risk (POTTER) calculator in emergency general surgery and emergency laparotomy paTients. J Am Coll Surg 232(6):912-919.e1. 10.1016/j.jamcollsurg.2021.02.009 33705983 10.1016/j.jamcollsurg.2021.02.009

[34] Fleisher LA, Fleischmann KE, Auerbach AD et al (2014) 2014 ACC/AHA guideline on perioperative cardiovascular evaluation and management of patients undergoing noncardiac surgery: executive summary: a report of the American College of Cardiology/American Heart Association Task Force on Practice Guidelines. Circulation 130(24):2215–2245. 10.1161/CIR.0000000000000105 25085962 10.1161/CIR.0000000000000105

[35] Ford MK, Beattie WS, Wijeysundera DN (2010) Systematic review: prediction of perioperative cardiac complications and mortality by the revised cardiac risk index. Ann Intern Med 152(1):26–35. 10.7326/0003-4819-152-1-201001050-00007 20048269 10.7326/0003-4819-152-1-201001050-00007

[36] Bihorac A, Ozrazgat-Baslanti T, Ebadi A et al (2019) MySurgeryRisk: development and validation of a machine-learning risk algorithm for major complications and death after surgery. Ann Surg 269(4):652–662. 10.1097/SLA.0000000000002706 29489489 10.1097/SLA.0000000000002706 PMC6110979

[37] Solanki SL, Kaur J, Gupta AM et al (2021) Cancer related nutritional and inflammatory markers as predictive parameters of immediate postoperative complications and long-term survival after hepatectomies. Surg Oncol 37:101526. 10.1016/j.suronc.2021.101526 33582497 10.1016/j.suronc.2021.101526

[38] Wang J, Zheng T, Liao Y et al (2022) Machine learning prediction model for post- hepatectomy liver failure in hepatocellular carcinoma: a multicenter study. Front Oncol 12:986867. 10.3389/fonc.2022.986867. (Published 2022 Nov 2) 36408144 10.3389/fonc.2022.986867 PMC9667038

[39] Connor CW, Segal S (2011) Accurate classification of difficult intubation by computerized facial analysis. Anesth Analg 112(1):84–93. 10.1213/ANE.0b013e31820098d6 21081769 10.1213/ANE.0b013e31820098d6

[40] Wang G, Li C, Tang F et al (2023) A fully-automatic semi-supervised deep learning model for difficult airway assessment. Heliyon 9(5):e15629. 10.1016/j.heliyon.2023.e15629 37159696 10.1016/j.heliyon.2023.e15629 PMC10163620

[41] Kendale S, Kulkarni P, Rosenberg AD, Wang J (2018) Supervised machine-learning predictive analytics for prediction of postinduction hypotension. Anesthesiology 129(4):675–688. 10.1097/ALN.0000000000002374 30074930 10.1097/ALN.0000000000002374

[42] Hatib F, Jian Z, Buddi S et al (2018) Machine-learning algorithm to predict hypotension based on high-fidelity arterial pressure waveform analysis. Anesthesiology 129(4):663–674. 10.1097/ALN.0000000000002300 29894315 10.1097/ALN.0000000000002300

[43] Gangakhedkar GR, Solanki SL, Divatia JV (2022) The use of Hypotension Prediction Index in cytoreductive surgery (CRS) with hyperthermic intraperitoneal chemotherapy (HIPEC). Indian J Anaesth 66(4):294–298. 10.4103/ija.ija_102_22 35663216 10.4103/ija.ija_102_22 PMC9159399

[44] Lee S, Lee HC, Chu YS et al (2021) Deep learning models for the prediction of intraoperative hypotension. Br J Anaesth 126(4):808–817. 10.1016/j.bja.2020.12.035 33558051 10.1016/j.bja.2020.12.035

[45] Veselis RA, Reinsel R, Sommer S, Carlon G (1991) Use of neural network analysis to classify electroencephalographic patterns against depth of midazolam sedation in intensive care unit patients. J Clin Monit 7(3):259–267. 10.1007/BF01619271 1890449 10.1007/BF01619271

[46] Ortolani O, Conti A, Di Filippo A et al (2002) EEG signal processing in anaesthesia. Use of a neural network technique for monitoring depth of anaesthesia. Br J Anaesth 88(5):644–648. 10.1093/bja/88.5.644 12067000 10.1093/bja/88.5.644

[47] Mirsadeghi M, Behnam H, Shalbaf R, Jelveh MH (2016) Characterizing awake and anesthetized states using a dimensionality reduction method. J Med Syst 40(1):13. 10.1007/s10916-015-0382-4 26573650 10.1007/s10916-015-0382-4

[48] Shalbaf A, Saffar M, Sleigh JW, Shalbaf R (2018) Monitoring the depth of anesthesia using a new adaptive neurofuzzy system. IEEE J Biomed Health Inform 22(3):671–677. 10.1109/JBHI.2017.2709841 28574372 10.1109/JBHI.2017.2709841

[49] Liu Q, Ma L, Fan SZ, Abbod MF, Shieh JS (2018) Sample entropy analysis for the estimating depth of anaesthesia through human EEG signal at different levels of unconsciousness during surgeries. PeerJ 6:e4817. 10.7717/peerj.4817. (Published 2018 May 23) 29844970 10.7717/peerj.4817 PMC5970554

[50] Nagaraj SB, Biswal S, Boyle EJ et al (2017) Patient-specific classification of ICU sedation levels from heart rate variability. Crit Care Med 45(7):e683–e690. 10.1097/CCM.0000000000002364 28441231 10.1097/CCM.0000000000002364 PMC5474145

[51] Solanki SL, Pandrowala S, Nayak A, Bhandare M, Ambulkar RP, Shrikhande SV (2021) Artificial intelligence in perioperative management of major gastrointestinal surgeries. World J Gastroenterol 27(21):2758–2770. 10.3748/wjg.v27.i21.2758 34135552 10.3748/wjg.v27.i21.2758 PMC8173379

[52] Moore BLMBL, Panousis P, Kulkarni V, Pyeatt L, Doufas AGDAG (2010) Reinforcement learning for closed-loop propofol anesthesia: a human volunteer study. Proc AAAI Conf Artif Intell 24(2):1807–1813. 10.1609/aaai.v24i2.18817

[53] Yang N, Yang M, Peng W, et al (2020) Comparison of anesthesia effects between closed-loop and open-loop target controlled infusion of propofol using the bispectral index in abdominal surgery. Journal of Central South University. Med Sci 45(12):1419–1424. 10.11817/j.issn.1672-7347.2020.190489 10.11817/j.issn.1672-7347.2020.190489 33472997

[54] Puri GD, Mathew PJ, Biswas I et al (2016) A multicenter evaluation of a closed-loop anesthesia delivery system: a randomized controlled trial. Anesth Analg 122(1):106–114. 10.1213/ANE.0000000000000769 25902324 10.1213/ANE.0000000000000769

[55] Avital G, Snider EJ, Berard D et al (2022) Closed-loop controlled fluid administration systems: a comprehensive scoping review. J Pers Med 12(7):1168. 10.3390/jpm12071168. (Published 2022 Jul 18) 35887665 10.3390/jpm12071168 PMC9315597

[56] Chen L, Dubrawski A, Wang D et al (2016) Using supervised machine learning to classify real alerts and artifact in online multisignal vital sign monitoring data. Crit Care Med 44(7):e456–e463. 10.1097/CCM.0000000000001660 26992068 10.1097/CCM.0000000000001660 PMC4911247

[57] Guillame-Bert M, Dubrawski A, Wang D, Hravnak M, Clermont G, Pinsky MR (2017) Learning temporal rules to forecast instability in continuously monitored patients. J Am Med Inform Assoc 24(1):47–53. 10.1093/jamia/ocw048 27274020 10.1093/jamia/ocw048 PMC5201181

[58] Rinehart J, Le Manach Y, Douiri H et al (2014) First closed-loop goal directed fluid therapy during surgery: a pilot study. Ann Fr Anesth Reanim 33(3):e35–e41. 10.1016/j.annfar.2013.11.016 24378044 10.1016/j.annfar.2013.11.016

[59] Rinehart J, Lilot M, Lee C et al (2015) Closed-loop assisted versus manual goal-directed fluid therapy during high-risk abdominal surgery: a case-control study with propensity matching. Crit Care 19(1):94. 10.1186/s13054-015-0827-7. (Published 2015 Mar 19) 25888403 10.1186/s13054-015-0827-7 PMC4372998

[60] Joosten A, Chirnoaga D, Van der Linden P et al (2021) Automated closed-loop versus manually controlled norepinephrine infusion in patients undergoing intermediate- to high-risk abdominal surgery: a randomised controlled trial. Br J Anaesth 126(1):210–218. 10.1016/j.bja.2020.08.051 33041014 10.1016/j.bja.2020.08.051 PMC8489152

[61] Joosten A, Rinehart J, Van der Linden P et al (2021) Computer-assisted individualized hemodynamic management reduces intraoperative hypotension in intermediate- and high-risk surgery: a randomized controlled trial. Anesthesiology 135(2):258–272. 10.1097/ALN.0000000000003807 33951140 10.1097/ALN.0000000000003807 PMC8277754

[62] Pesteie M, Lessoway V, Abolmaesumi P, Rohling RN (2018) Automatic localization of the needle target for ultrasound-guided epidural injections. IEEE Trans Med Imaging 37(1):81–92. 10.1109/TMI.2017.2739110 28809679 10.1109/TMI.2017.2739110

[63] Hetherington J, Lessoway V, Gunka V, Abolmaesumi P, Rohling R (2017) SLIDE: automatic spine level identification system using a deep convolutional neural network. Int J Comput Assist Radiol Surg 12(7):1189–1198. 10.1007/s11548-017-1575-8 28361323 10.1007/s11548-017-1575-8

[64] Zhao Y, Zheng S, Cai N et al (2023) Utility of artificial intelligence for real-time anatomical landmark identification in ultrasound-guided thoracic paravertebral block. J Digit Imaging 36(5):2051–2059. 10.1007/s10278-023-00851-8 37291383 10.1007/s10278-023-00851-8 PMC10501964

[65] Bowness JS, Burckett-St Laurent D, Hernandez N et al (2023) Assistive artificial intelligence for ultrasound image interpretation in regional anaesthesia: an external validation study. Br J Anaesth 130(2):217–225. 10.1016/j.bja.2022.06.031 35987706 10.1016/j.bja.2022.06.031 PMC9900723

[66] Alkhatib M, Hafiane A, Vieyres P, Delbos A (2019) Deep visual nerve tracking in ultrasound images. Comput Med Imaging Graph 76:101639. 10.1016/j.compmedimag.2019.05.007 31349184 10.1016/j.compmedimag.2019.05.007

[67] Oh TT, Ikhsan M, Tan KK et al (2019) A novel approach to neuraxial anesthesia: application of an automated ultrasound spinal landmark identification. BMC Anesthesiol 19(1):57. 10.1186/s12871-019-0726-6. (Published 2019 Apr 16) 30991949 10.1186/s12871-019-0726-6 PMC6469214

[68] Lucey P, Cohn JF, Matthews I et al (2011) Automatically detecting pain in video through facial action units. IEEE Trans Syst Man Cybern B Cybern 41(3):664–674. 10.1109/TSMCB.2010.2082525 21097382 10.1109/TSMCB.2010.2082525 PMC6942457

[69] Behrman M, Linder R, Assadi AH, Stacey BR, Backonja MM (2007) Classification of patients with pain based on neuropathic pain symptoms: comparison of an artificial neural network against an established scoring system. Eur J Pain 11(4):370–376. 10.1016/j.ejpain.2006.03.001 16624601 10.1016/j.ejpain.2006.03.001

[70] Lopez-Martinez D, Eschenfeldt P, Ostvar S, Ingram M, Hur C, Picard R (2019) Deep reinforcement learning for optimal critical care pain management with morphine using dueling double-deep Q networks. Annu Int Conf IEEE Eng Med Biol Soc 2019:3960–3963. 10.1109/EMBC.2019.8857295 31946739 10.1109/EMBC.2019.8857295

[71] Wang Q, Yuan L, Ding X, Zhou Z (2021) Prediction and diagnosis of venous thromboembolism using artificial intelligence approaches: a systematic review and meta-analysis. Clin Appl Thromb Hemost 27:10760296211021162. 10.1177/10760296211021162 34184560 10.1177/10760296211021162 PMC8246532

[72] Ambra N, Mohammad OH, Naushad VA et al (2022) Venous thromboembolism among hospitalized patients: incidence and adequacy of thromboprophylaxis - a retrospective study. Vasc Health Risk Manag 18:575–587. 10.2147/VHRM.S370344. (Published 2022 Jul 24) 35912018 10.2147/VHRM.S370344 PMC9333096

[73] Zhou S, Ma X, Jiang S, Huang X, You Y, Shang H, Lu Y (2021) A retrospective study on the effectiveness of Artificial Intelligence-based Clinical Decision Support System (AI-CDSS) to improve the incidence of hospital-related venous thromboembolism (VTE). Annals Of Translational Medicine 9(6):491. 10.21037/atm-21-1093 33850888 10.21037/atm-21-1093 PMC8039638

[74] Adams J (2023) Defending explicability as a principle for the ethics of artificial intelligence in medicine. Med Health Care Philos 26(4):615–623. 10.1007/s11019-023-10175-7 10.1007/s11019-023-10175-7 PMC10725847 37642834

[75] Herington J, McCradden MD, Creel K et al (2023) Ethical considerations for artificial intelligence in medical imaging: deployment and governance. J Nucl Med 64(10):1509–1515. 10.2967/jnumed.123.266110 37620051 10.2967/jnumed.123.266110 PMC12782051

